# Spontaneous assemblies of gigantic polyoxomolybdates; from structure and properties to synthetic methods

**DOI:** 10.1039/d5dt03084f

**Published:** 2026-03-11

**Authors:** Vishal Lakhanpal, De-Liang Long, Leroy Cronin

**Affiliations:** a Advanced Research Centre (ARC), Level 5, Digital Chemistry, University of Glasgow 11 Chapel Lane Glasgow G11 6EW UK Lee.Cronin@glasgow.ac.uk

## Abstract

Gigantic polyoxomolybdates, dominated by the Molybdenum Blue (MB) family, constitute a key subclass of polyoxometalates (POMs) and play a fundamental role in elucidating structural construction principles and showcasing the diversity of POM architectures. Since the first structural determination of the iconic {Mo_154_} big wheel cluster, these giant polyoxomolybdates have been developed rapidly over the past three decades, emerging as one of the most versatile classes of POM clusters. Potential applications span materials science, electronic materials, and catalysis. This tutorial review summarises the fundamentals of MBs and their close relatives including the highly reduced Mo browns and Mo reds, their molecular and electronic structures, physical properties, and synthetic methods for production. Drawing on 67 references from pivotal studies over the last thirty years, this review serves as both an authoritative reference for early-career researchers and an accessible introduction for non-professionals interested in this dynamic field.

## Abstract 2

Gigantic polyoxomolybdates, dominated by the Molybdenum Blue (MB) family, constitute a key subclass of polyoxometalates (POMs) and play a role in elucidating structural construction principles and showcasing the diversity of POM architectures.

## Graphical abstract

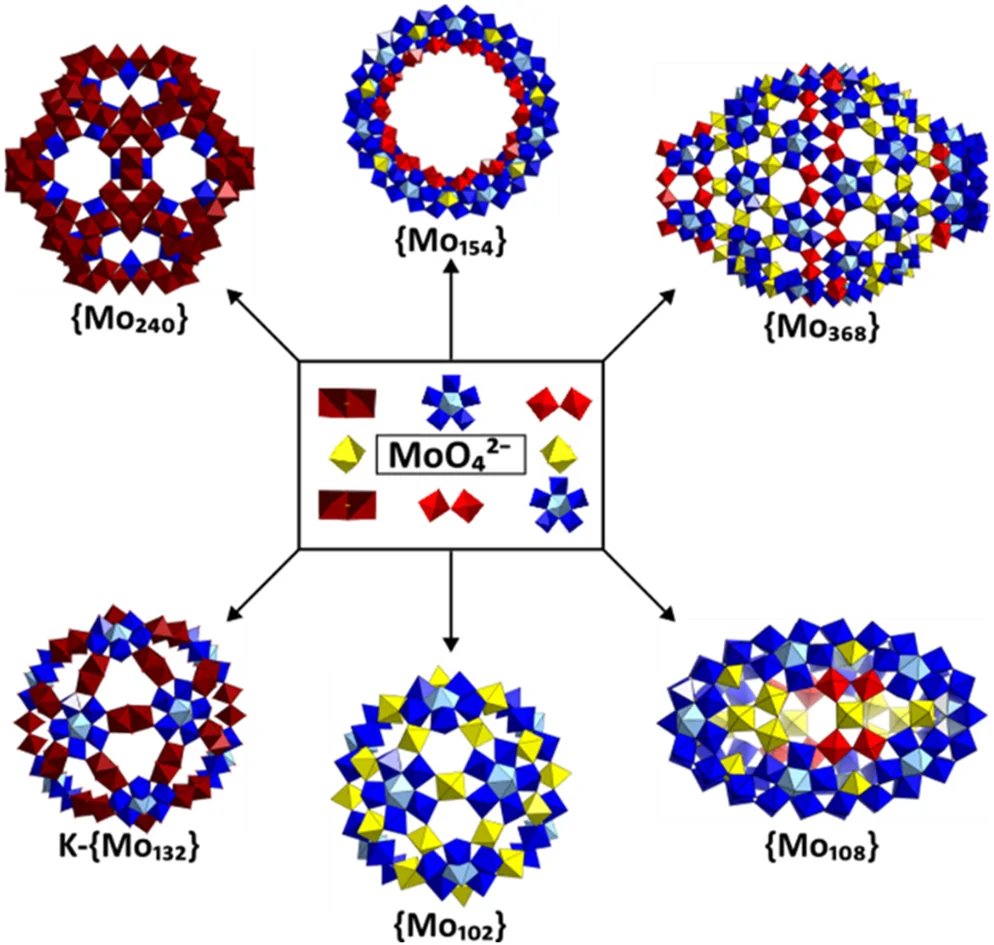

**Key learning points**

(1) The generation of polyoxomolybdate building blocks and their associated structures.

(2) Parameters for synthetic control governing cluster self-assembly.

(3) The electronic structure of giant polyoxomolybdates.

(4) Their properties and potential applications.

(5) Synthetic procedures and characterisation methods for isolation and analysis.

## 1. Introduction

Polyoxometalates (POMs) are discrete inorganic metal oxide clusters composed of Group V (V, Nb, Ta) or Group VI (Mo, W) elements in their highest oxidation states.^1–6^ These remarkable molecules exhibit exceptional structural complexity, self-assembling from simple metal salt solutions to yield a diverse array of architectures with rich chemical and physical properties. Their compositional and structural versatility underpins their widespread applications, including catalysis, electronic devices, and functional materials, leveraging their redox, spectroscopic, photovoltaic, and magnetic characteristics.^7–15^ A distinct subgroup of POMs consists of gigantic, ‘nanosized’ polyoxomolybdate (POMo) clusters, most notably molybdenum blues (MBs).

After three decades of development, gigantic POMos have become a central focus of POM chemistry, driving advances in both fundamental understanding and practical applications.^16–23^ To date, over one hundred POMo species have been discovered and structurally characterised. Table 1 provides the full chemical formulae of representative POMos discussed in detail throughout this tutorial review; within main text, these species are described using abbreviated notations for clarity and consistency.

**Table 1:** Giant POMo architectures, grouped by architecture, with full formulae

Architecture	Notation	Composition	Ref.
Blue wheel	{Mo_138_}	[Mo^VI^_110_Mo^V^_28_O_416_H_6_(H_2_O)_58_(CH_3_CO_2_)_6_]^32−^	36
Blue wheel	{Mo_154_}	[Mo^VI^_126_Mo^V^_28_O_462_H_14_(H_2_O)_70_]^14−^	25
Blue wheel	{Mo_176_}	[Mo^VI^_144_Mo^V^_32_O_528_H_16_(H_2_O)_80_]^16−^	26
Blue wheel	{Mo_90_Ln_10_}	[Mo^VI^_70_Mo^V^_20_Ln_10_O_280_H_10_(H_2_O)_80_] (Ln = La, Ce and Pr)	31
Blue wheel	{Mo_120_Ln_6_}	[Mo^VI^_96_Mo^V^_24_Ln_6_O_366_H_12_(H_2_O)_78_]^6−^ (Ln = Ce, Pr)	37 and 38
Blue ellipsoid	{Mo_85_}	[Mo^VI^_61_Mo^V^_18_Mo^IV^_6_H_48_O_267_S_2_(SO_3_)_3_]^16−^	34
Blue ellipsoid	{Mo_108_}	[Mo^VI^_84_Mo^V^_24_H_78_O_358_]^14−^	34
Blue cage	L-{Mo_132_}	[H_30_ Mo^VI^_92_Mo^V^_10_Mo^IV^_30_(H_2_O)_36_O_382.4_S_9.6_(SO_4_)_2_]^36−^	32
Green cage	C-{Mo_132_}	[Mo^VI^_72_Mo^V^_60_O_372_(OH)_10_(H_2_O)_12_(SO_4_)_5_(CH_3_COO)_20_]^52−^	33
Blue cage	{Mo_248_}	[Mo^VI^_168_Mo^V^_80_O_720_H_16_(H_2_O)_128_]^16−^	27
Blue ball	{Mo_102_}	[Mo^VI^_66_Mo^V^_36_(H_2_O)_78_(CH_3_CO_2_)_12_]	28
Brown ball	K-{Mo_132_}	[Mo^VI^_72_Mo^V^_60_(H_2_O)_72_(CH_3_CO_2_)_30_]^42−^	29
Red ball	{Mo_240_}	[Mo^VI^_60_Mo^V^_180_O_680−*x*_H_60_(SO_3_)_20−*x*_(SO_4_)_*x*_]^(80−2*x*)−^	35
Red cube	{Mo_64_}	[Mo^VI^_12_Mo^V^_52_H_26_O_200_]^42−^	39
Red star	{Mo_70_}	[Mo^VI^_30_Mo^V^_40_H_30_O_215_]^20−^	39
Blue lemon	{Mo_368_}	[Mo^VI^_256_Mo^V^_112_O_1032_H_16_(H_2_O)_240_(SO_4_)_48_]^48−^	24

## 2. Gigantic polyoxomolybdate structures

### 2.1. Structural diversity

Gigantic POMos are species that far exceed the sizes of regular POMs, ranging from the smallest member with only 36 Mo centres to the largest, protein-sized cluster with 368 Mo atoms.^24^ This remarkable size diversity enables POMos to exhibit properties that bridge the gap between well-defined molecular entities and less structured polymeric systems. Within this classification lies a number of architectures, spanning the sub types known as Mo blue, Mo brown and Mo red, so called because of the degree of reduction of the cluster. Gigantic POMos experienced rapid advancement following the groundbreaking discovery of the giant wheel-shaped {Mo_154_} in 1996.^25^ The structure was obtained through the crystallisation of a reduced, acidified blue molybdate solution, which when left undisturbed-yielded single crystals suitable for X-ray analysis. This work marked the first structural characterisation of a super large POMo and paved the way for the synthesis of numerous related clusters.

**Fig. 1:**
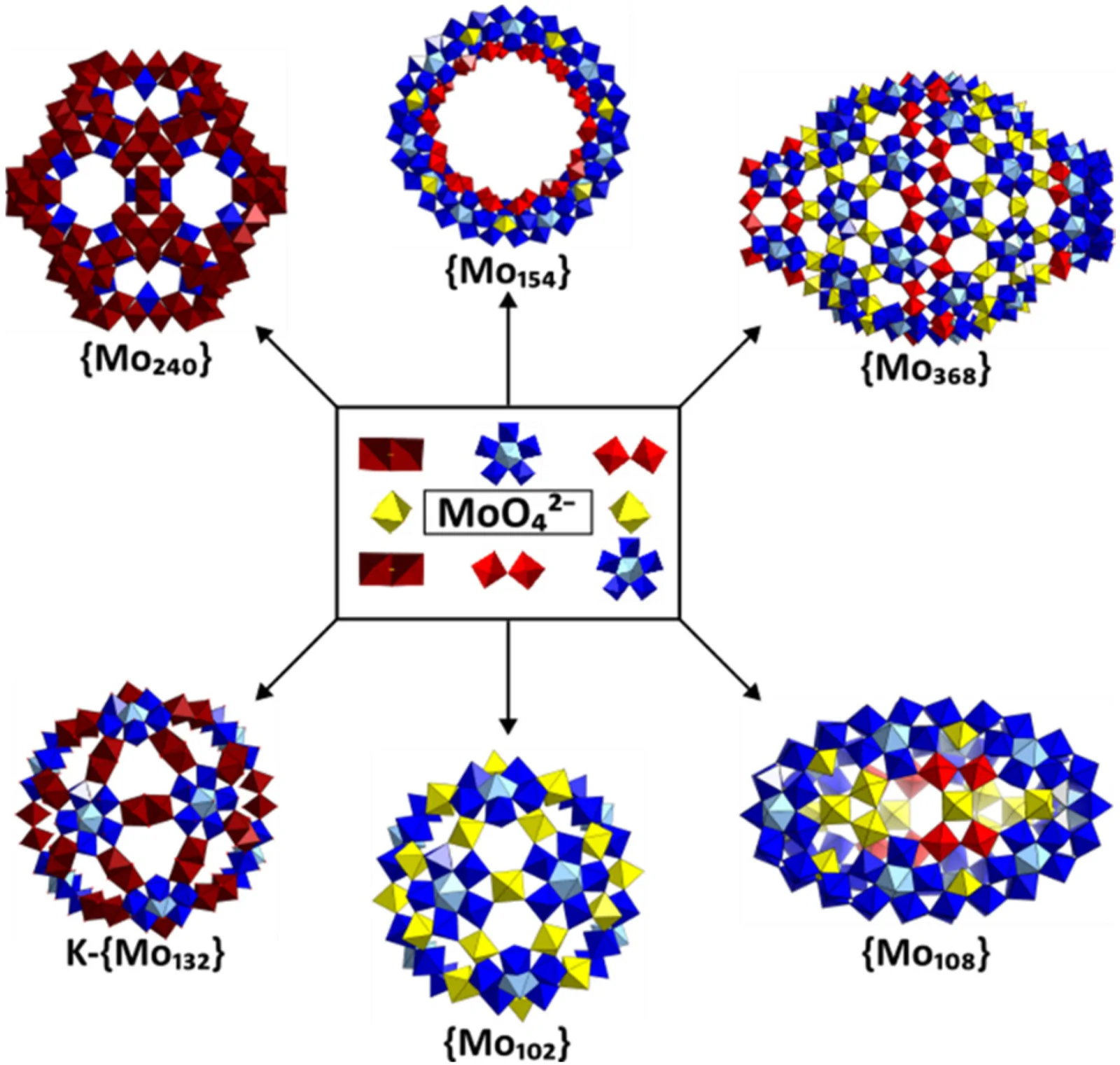
Polyhedral depiction of discrete BBs which produce a range of POMo clusters, depicted are {Mo_154_}, {Mo_368_}, {Mo_108_}, {Mo_102_}, K-{Mo_132_} and {Mo_240_}.

Focusing specifically on the MB family, several teams have since developed a diverse array of architectures, evolving from simple ring structures to more complex geometries.^24–27^ Subsequent studies expanded the family of MBs and led to the discovery of analogous structures, some featuring higher degrees of Mo centre reduction, namely Mo browns and reds. These include analogue wheel-shaped {Mo_176_},^26^ the spherical {Mo_102_},^28^ Keplerate ball K-{Mo_132_},^29^ capped-wheel {Mo_248_},^27^ and lemon-shaped {Mo_368_}^24^ clusters (Fig. 1), each exhibiting distinct structural and functional characteristics. More recent advances by other researchers extended to the discovery of the half expanded wheel {Mo_180_},^30^ neutral wheel shaped {Mo_90_Ln_10_},^31^ the lantern shaped L-{Mo_132_},^32^ capped wheel C-{Mo_132_},^33^ compressed cycle {Mo_108_}^34^ and Mo red {Mo_240_}^35^*etc*. These recent advances highlight the structural diversity of POMos, whilst also providing insight into the capability of these large-scale assemblies to display quasi-isomerism *i.e.* K-{Mo_132_}, L-{Mo_132_}, and C-{Mo_132_}. This pseudo-isomerism refers to the identical nuclearity of Mo centres; however, it does not constitute true isomerism as these species differ in their number of oxygen and heteroatoms present (Table 1).

### 2.2. Building blocks

**Fig. 2:**
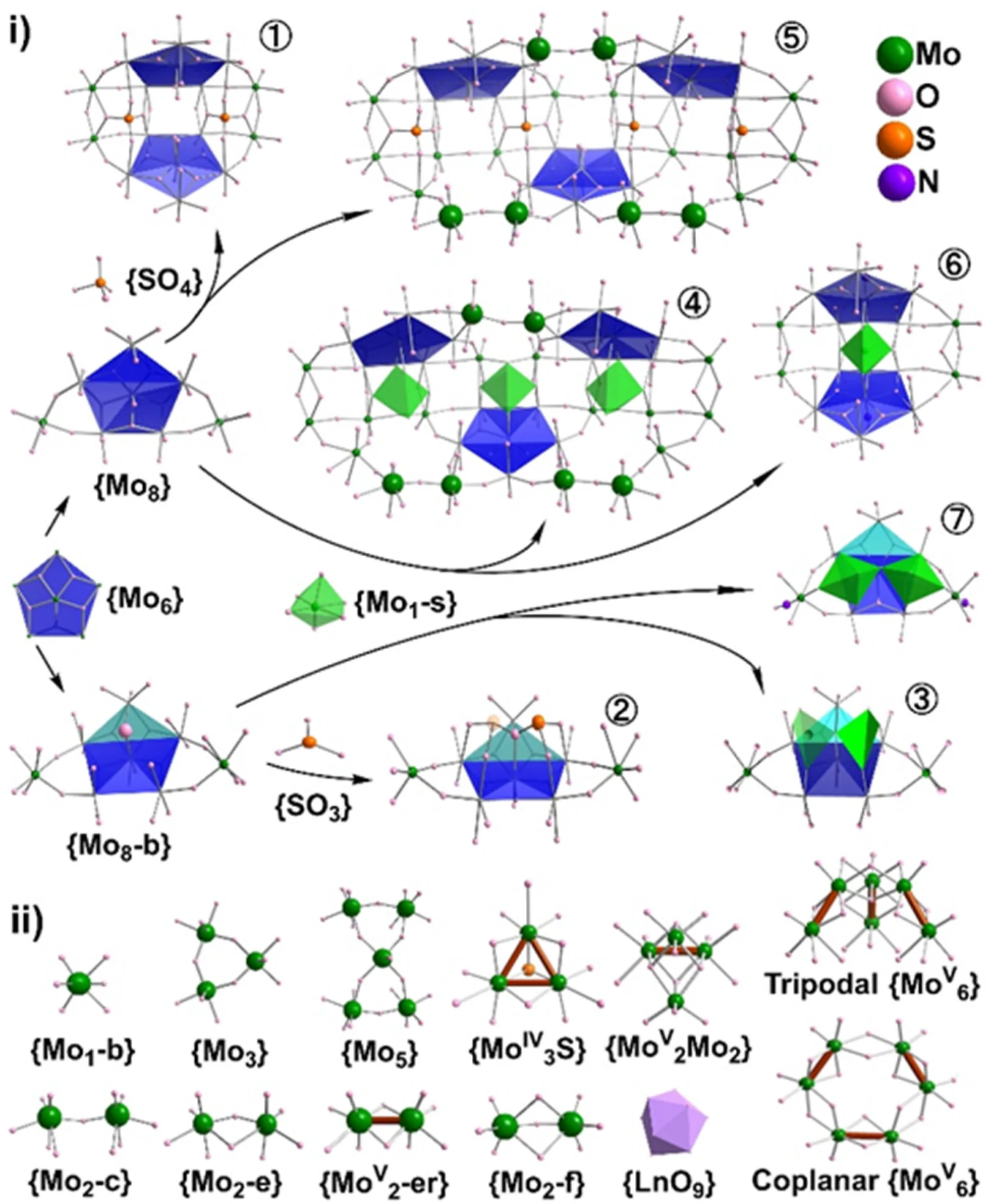
Building blocks giving rise to common motifs and virtual building blocks. (i) {Mo_6_}, {Mo_8_} and {Mo_8_-b} building block. {Mo_8_-b} possesses an out of plane shared oxo ligand (larger sphere), distorting the building block. Seven MB construction schemes as supporting modes ①–⑦ of supporting units {SO_4_}, {SO_3_} and {Mo_1_-s} to pentagon units. (ii) Bridging units typically observed in POMo assemblies {Mo^IV^_3_S}, corner sharing {Mo_2_-c}, edge-sharing {Mo_2_-e}, reduced edge sharing {Mo_2_-er}, face sharing {Mo_2_-f}, bridging {Mo_5_} and lanthanide ion {LnO_9_};^34^ cubic {Mo^V^_2_Mo_2_}, tripodal {Mo^V^_6_} and coplanar {Mo^V^_6_} for constructions of Mo reds.

Most POMos (MBs and Mo browns) share common structural features, utilising transferable pentagonal {Mo_6_} or derived {Mo_8_} groups as fundamental building blocks (BBs), along with variable supporting/templating and bridging units. Fig. 2 summarises these building units and their assembly schemes through different templating modes. The pentagonal BB is key to promoting molecular growth of MB architectures. This arises due to the ability of the key {Mo_6_} BB to form ‘virtual building blocks’ (VBB), which are common motifs that are not usually discrete observable units but can be seen in the overall structures. The archetypal {Mo_154_}^25^ and {Mo_176_}^26^ wheel structures consist of 14 or 16 standard pentagon-based {Mo_8_} BB, respectively. These are connected by an equal number of supporting {Mo_1_-s} units, forming the structural backbone and {Mo_2_-c} bridging units create the wheel's outer edge. The wheel size is determined by the number of pentagonal {Mo_6_} and {Mo_2_-c} units. The fixed dimension requirement of {Mo_2_-c} units for ring completion explains the limited accessibility of other wheel structures beyond {Mo_154_} and {Mo_176_} at initial stages of MB exploration. Following studies have demonstrated that smaller MB clusters can be obtained by partially or completely replacing {Mo_2_-c} units with smaller bridging units, such as lanthanide (Ln) or uranyl ions. Examples include {Mo_128_Ln_4_},^40^ {Mo_124_Ln_4_},^41–44^ {Mo_120_Ln_6_},^37,38^ {Mo_100_Ln_6_},^45^ {Mo_90_Ln_10_},^31,33^ and {Mo_90_U_10_},^46^ which contain 12 or 10 {Mo_8_} BBs. More recent advances show that substituting {Mo_2_-c} units with smaller {Mo_2_-er} units produce the capped-wheel C-{Mo_132_} cluster.^33^ This MB-type structure contains a circular array of 10 {Mo_8_} pentagonal BB, each of which features a {Mo_2_-er} unit (Fig. 2) that extends at the outer-most apex laterally within the pentagon's plane, directed toward the cluster's C_5_ main axis. These in-plane {Mo_2_-er} extensions function as connecting beams, linking to two {Mo_6_} pentagonal units that cap opposite ends of the C-{Mo_132_} assembly. The lantern shaped L-{Mo_132_}^32^ also contain 10 {Mo_8_} pentagonal BBs on the backbone/belt. 10 {Mo_3_S} triads replace the {Mo_2_-c} units on ring edges, acting as bridging units to connect two {Mo_6_} pentagonal units which cap opposite ends of the cluster to form a closed shell structure.

The {Mo_102_} cluster represents another MB-type structure but adopts a distinct spherical architecture.^28^ Its framework comprises 30 {Mo_1_-b} bridging units that interconnect 12 {Mo_6_} pentagons into a complete ball-shaped structure. Similarly, the ball-shaped Keplerate K-{Mo_132_}^29^ (a Mo brown variant) follows an analogous construction pattern to {Mo_102_}, but with 30 {Mo_2_-er} units occupying the {Mo_1_-b} positions. Remarkably, K-{Mo_132_}, L-{Mo_132_}^32^ and C-{Mo_132_} contain equal metal nuclearity, but their skeleton symmetries are significantly different. While K-{Mo_132_} is a regular ball with icosahedral symmetry, L-{Mo_132_} and C-{Mo_132_} feature rare oblate spheroids of *D*_5d_ symmetry.

The molecular growth of MB clusters can be further controlled,^47^ where the wheels display^51,53,55^ the capability of being compressed towards smaller ring or ellipsoidal architectures.^34^ It has been observed that the pentagonal {Mo_8_} units can adopt two conformations, one of which is shared with traditional MB architectures where all Mo atoms are planar; the other adopted conformation is a distorted arrangement where one peak of the pentagon lies out of the plane, {Mo_8_-b}. The distortion allows for the MB species formed to adopt new compressed architectures because of a unique bonding mode of {Mo_8_-b} units involved in MB backbone construction *via* the distorted peak. This arrangement leads to a total loss of horizontal pseudo mirror symmetry of traditional wheel architectures.

**Fig. 3:**
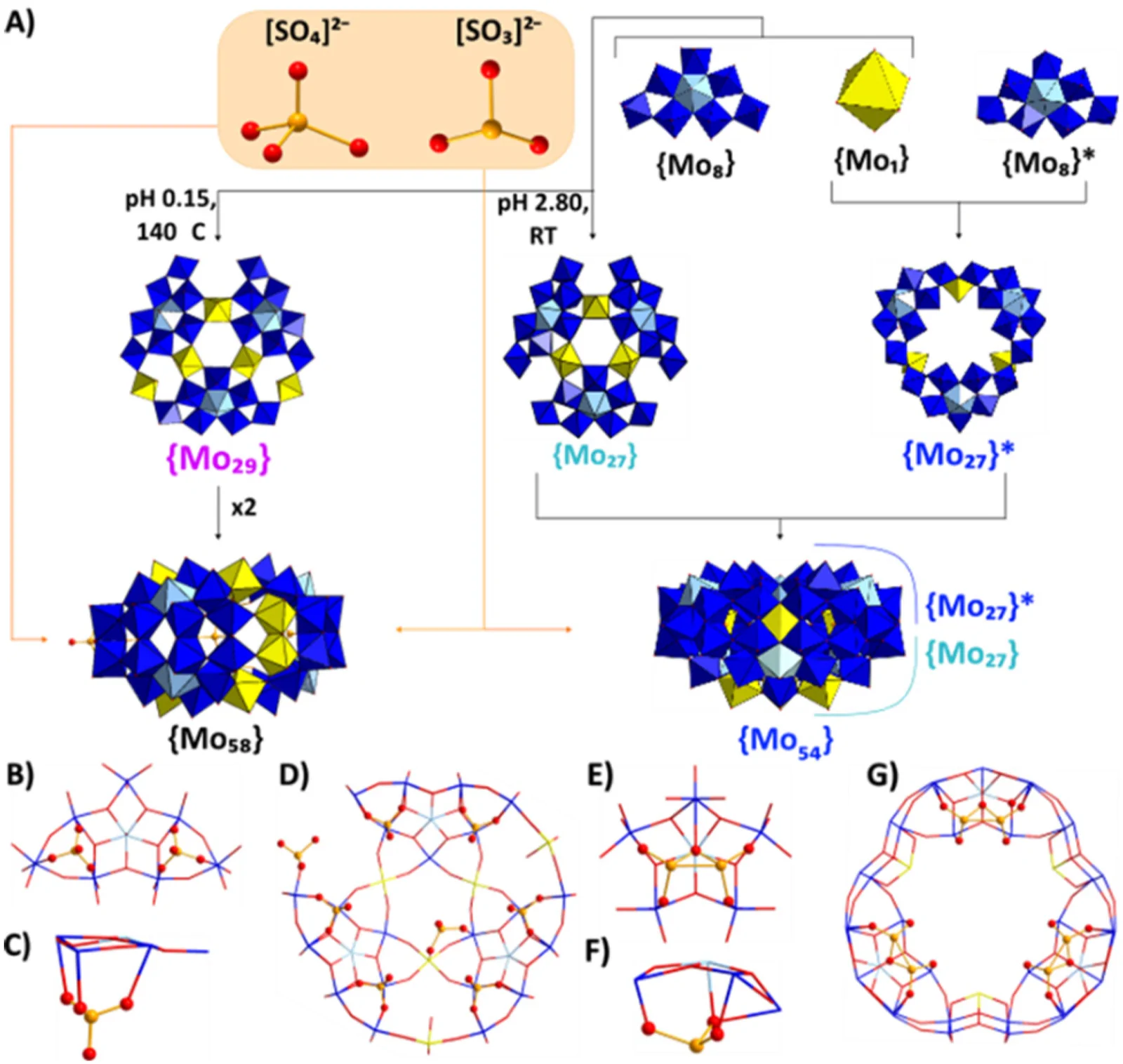
(A) Schematic representation of the formation of {Mo_54_} and {Mo_58_}. Mo^VI^: blue polyhedra. Mo^V^: yellow or blue polyhedra. Further indicated are the templating hetero-anionic species, SO_3_^2−^ and SO_4_^2−^ where the former templates both clusters whilst the latter only templates in {Mo_54_}. (B) and (E) indicate the bonding off the templates within the pentagonal units, (C) and (F) indicate the geometric arrangement of the BB due to the hetero-anionic templates whilst (D) and (G) display the resultant POM architecture by the arrangement of the BB. Note that one sulphur atom (orange) is disordered over two positions each with 50% occupancy species.

The compression of MB wheels from 154 Mo atoms to 54, producing {Mo_54_},^34^ was achieved by employing distort pentagonal {Mo_8_-b} BB. {Mo_54_} represents the smallest MB consisting only six pentagonal BB and displays a bowl-shaped architecture (Fig. 3). The distorted pentagonal {Mo_8_-b} BB were found to be crucial for constructing other non-traditional MBs such as {Mo_85_} and {Mo_108_}, which display the shape of a flattened American football. Further related structures could be made by introducing hetero species such as {SO_3_}, {SO_4_}, {RPO_3_} and carboxylate ligands, which can exist as templating or bridging units to alter the architectures. Within a given MB POM where a ratio of Mo^VI^ : Mo^V^ exists, the reducing electrons are typically delocalised. The delocalisation of the reducing electrons across the MB framework gives rise to the characteristic blue colour, where these clusters display an absorption peak at approximately 750 nm in the UV-vis-NIR electronic spectra.^39^ The brown and red colours of the more reduced POMos arises from the localisation of reducing electrons on the Mo–Mo bonding, unlike MB counterparts. Table 2 summarises the properties of representative giant POMos, in which MBs with reduction ratio (reducing electrons/total Mo) from delocalised reducing electrons in descending order (highlighted in blue) and Mo red with reduction ratio from localised reducing electrons in ascending order (highlighted in red). In the middle, Mo brown K-{Mo_132_} and green C-{Mo_132_} are listed as their reduction ratios by localised reducing electrons are lower. It can be seen that total reduction ratios for MBs are generally lower than 50% while those for Mo reds are higher than 50%.

**Table 2:** Summary of giant POMos with their reduction ratios and the colours of the species

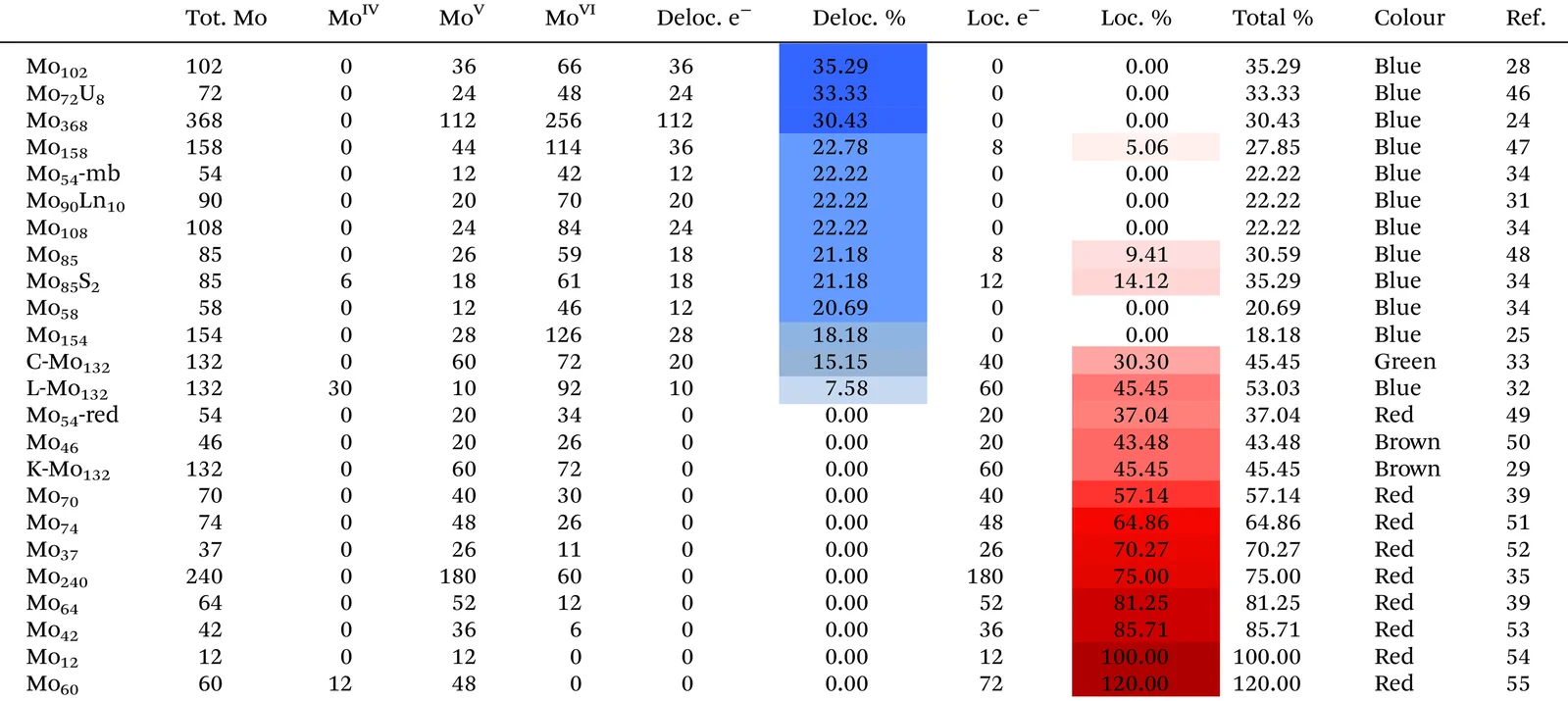

### 2.3. Molybdenum blues

When considering the broad categories of Mo blues, browns, and reds there exist a breadth of structure archetypes. Within the category of MB POMs, the classical archetype is the giant wheel framework, represented by {Mo_154_}^25^ the first giant MB POM identified. As described in previous section, {Mo_154_} is composed of pentagonal {Mo_6_}, corner sharing {Mo_2_-c}, and backbone supporting {Mo_1_-s} BBs. The {Mo_6_} units are distributed over both faces of the wheel, in a staggered base-to-base manner forming the MB backbone motif.

Previous studies have demonstrated that the nuclearity, and thus BB occupation on the MB wheel, can be controlled through pH adjustment during the synthetic procedure. Precise pH regulation enables directed assembly of MB wheel by modifying connective modes and selectively removing certain BBs from the framework. This control mechanism operates through pH-dependent charge modulation: increasing pH decreases negative charge in intermediary species, thereby lowering the nucleophilicity and diminishing the contribution of {Mo_2_-c} BBs. Ozeki *et al.*^56^ systematically demonstrated this phenomenon, showing that increasing the pH from 1.4 to 4.5 produced a series of MB rings ranging from {Mo_152_} to {Mo_138_}, with progressive loss of both {Mo_2_-c} and {Mo_1_-b} BBs. The deviation from the traditional 154 Mo membered ring was also accompanied by significant changes in the supramolecular organisation. At low pH, 1.4, discrete {Mo_150_} rings were observable alongside 1D {Mo_152_} chains, shifting towards 3D frameworks of {Mo_148_} at pH 1.7. This then further shifted towards 2D layering of {Mo_138_} at pH's ranging from 3.0 to 4.5. Notably, nuclearity of the ring structures can be controlled by means beyond pH control. By using entirely different synthetic methods, Cronin *et al.*^34^ have compressed the ring structures from 154 Mo atoms to 54, producing 54, 58, 85 and 108 Mo membered rings. This was achieved by utilising a mix of reducing agents in a given synthesis, coupled with hydrothermal heating conditions.^34^

Beyond compression of the tradition 154 membered ring, the nuclearity of ring species can also be expanded to {Mo_176_}:^26^ a 176 Mo membered ring, comprised of the same BBs found in the archetypal {Mo_154_}.^26^ This molecular expansion from {Mo_154_} to {Mo_176_} results in a subsequent increase in cavity size, with the larger {Mo_176_} ring possesses a 3 nm internal cavity. This cavity has been strategically utilised to facilitate further molecular growth, transforming the 176 Mo membered wheel into a 248 Mo membered cage.^27^ The {Mo_248_} cage possesses the fundamental characteristics of the {Mo_176_} framework but also incorporates {Mo_36_} capping units on both faces of the wheel. The capping process involves a condensation driven growth mechanism, originating at terminal oxo ligands of the internal {Mo_2_-c} BB skirt ring, which exhibit high proton affinity. As the interconnectivity between the caps and wheel are localised to the terminal oxo ligands of the {Mo_2_-c} BBs, the {Mo_36_} capping units are orientated at 22.5 degrees to each other.^26^

Beyond giant wheels, and the associated cage architectures, MB POMs have also been observed to adopt alternative architectures such as the spherical ball or the combination of where-ball curvature to give a ‘lemon’ shaped cluster with both positive and negative curvature. The most well-known ball framework is the {Mo_102_} MB cluster.^28^ This cluster closely resembles the classical Mo Brown Keplerate, K-{Mo_132_},^29^ where in both ball architectures are comprised of pentagonal {Mo_6_} BBs, interconnected by {Mo_1_-b} or {Mo_2_-er} linking units as described in the previous section.^28^

As described above, the lemon-shaped {Mo_368_}^24^ architecture can be viewed as a hybrid structure combining {Mo_176_} and {Mo_102_} motifs, demonstrating the remarkable potential for molecular growth of MB clusters.^24^ This giant cluster, approaching protein-sized dimensions with a 6 nm diameter, is assembled from 64 {Mo_1_-b}, 32 {Mo_2_-c} and 40 {Mo_6_} BBs. The cluster can be understood as a central {Mo_288_} spherical core flanked by two {Mo_40_} capping units at opposite ends. The architecture has an interesting symmetry with a central core that adopts *D*_8d_ symmetry whilst the caps display *C*_4v_ symmetry. The {Mo_6_} BBs within the structure can be further categorised into two types: those coordinated with SO_4_^2−^ anions and those uncoordinated. Coordinated {Mo_6_} BBs dominate the construction of {Mo_368_}, with 40 participating in the structure compared to only eight uncoordinated variants, participating in the structure. The hybrid nature of {Mo_368_} emerging from the diverse bonding behaviours of its BBs. The pentagonal {Mo_6_} units exhibit either extended construction patterns or adopt bonding modes of either spherical or wheel-like structures. This is exemplified by the presence of {Mo_11_} VBBs like those found in the {Mo_102_} cluster.^28^

### 2.4. Molybdenum browns and reds

Beyond the giant MB POMs, there also exist closely related counterparts: Mo browns and Mo reds. These categories are named based on the distinctive colours of their crystalline products, akin to the naming convention for Mo blues. The Mo brown and red variants exhibit different colours due to their greater degree of reduction alongside localised reducing electrons.

**Fig. 4:**
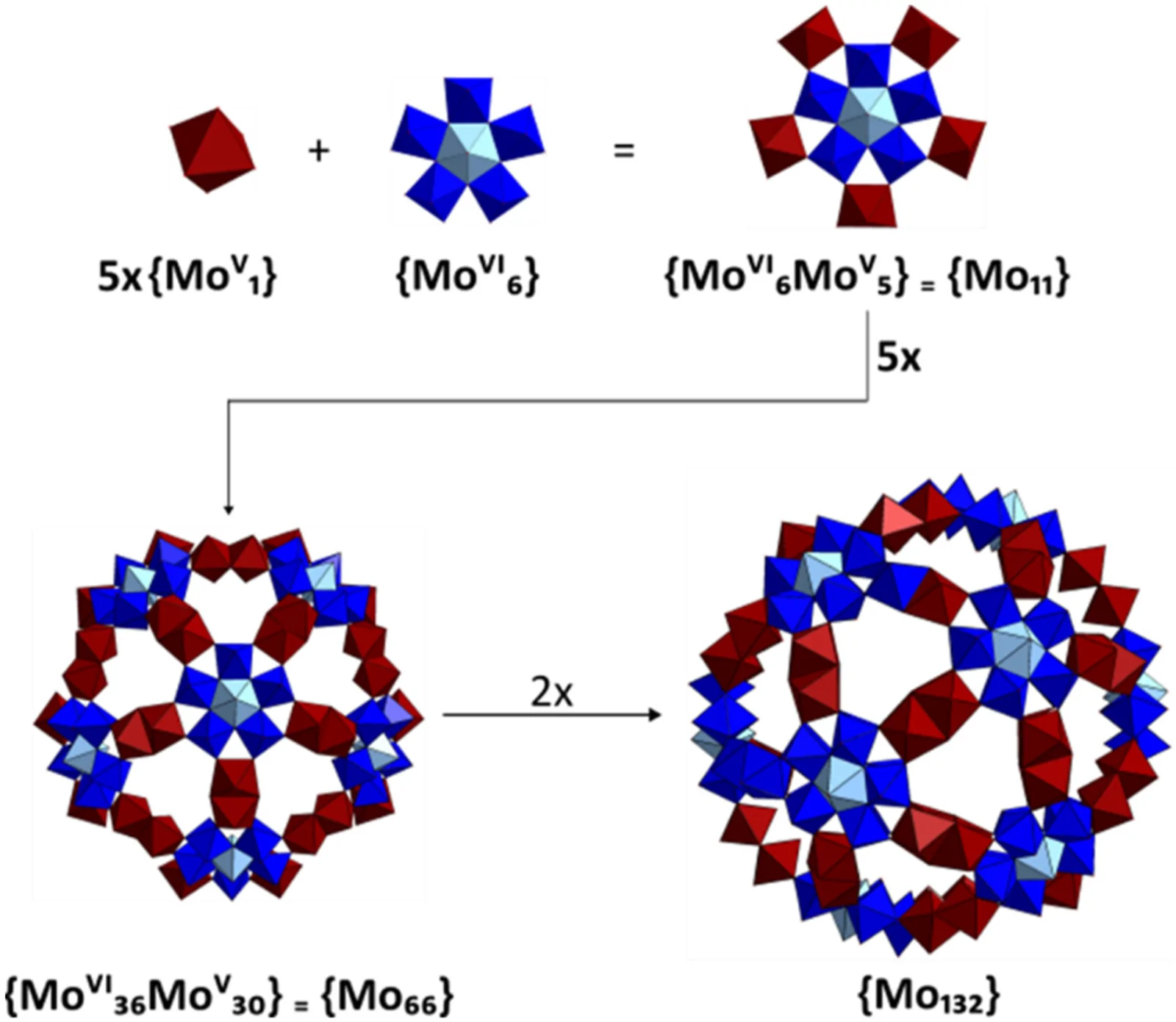
Schematic view of K-{Mo_132_} construction, proceeding from BB to VBB. Highlighted initially is the formation of the {Mo_11_} BB from the assembly of discrete {Mo^VI^_6_} and “virtual” {Mo^V^_1_}, followed by the formation of a {Mo_66_} VBB – which doubles to form {Mo_132_}.

Although Mo browns and reds represent a smaller subset of clusters, they possess unique structural features, making them a particularly interesting subfield within giant POMo chemistry. Among these, some giant clusters retain structural motifs from giant MB POMs and smaller classical POMs. A prominent example is the Mo brown Keplerate, K-{Mo_132_},^29^ which adopts a ball architecture. It consists of two types of BBs: {Mo_6_} and {Mo^V^_2_-er}. The arrangement of these units’ mimics that of {Mo_102_} – the pentagonal {Mo_6_} BBs bind with the {Mo^V^_2_} units in a corner sharing mode, where the {Mo^V^_2_} BBs act as linkers between adjacent {Mo_6_} units (Fig. 4). The {Mo^V^_2_} units are stabilised by bidentate acetate ligands, which are capable of undergoing exchange with other species such as sulphate, phosphate, succinate or glutarate.^57^

Lesser-known Mo browns include the chain-like {Mo_46_} cluster, a 46 Mo membered centrosymmetric cyclic structure.^50^ This unique assembly consists of two {Mo_21_} half-sections connected in a head-to-tail configuration by two bridging {Mo^V^_2_} units. Each {Mo_21_} segment incorporates chain-linked {Mo^V^_2_Mo^VI^_6_}, {Mo^V^_4_Mo^VI^_5_}, and {Mo^V^_2_Mo^VI^_2_} BBs, with structural reinforcement provided by two acetate bridging ligands at the junction points. The cluster contains 20 Mo^V^ centres, corresponding to a reduction ratio of 43.5%. All constituent BBs exhibit irregular geometries, with reducing electrons exclusively localised at Mo–Mo bonds. These characteristics clearly identify {Mo_46_} as a classic example of a Mo brown.

Moving beyond Mo browns, a further reduced classification of giant POMos are the Mo reds. The {Mo_37_}^52^ cluster is one such cluster, formerly classified as a Mo brown, composed of a central Keggin {Mo^V^_12_},^54^ capped by four {Mo^V^O_3_} units thus generating a {Mo^V^_16_} core. This is flanked by two similar but non-identical ligands: {Mo_11_} and {Mo_10_}. Two ligands differ with respect to the number of Mo centres (10 or 11) and the degree of reduction and protonation. The cluster contains 26 Mo^V^ centres, corresponding to a reduction ratio of *ca*. 70%. The {Mo_11_} and {Mo_10_} ligands exhibit irregular geometries, thus the whole cluster possesses asymmetric geometry. The reducing electrons are found localised at Mo–Mo bonds only.

**Fig. 5:**
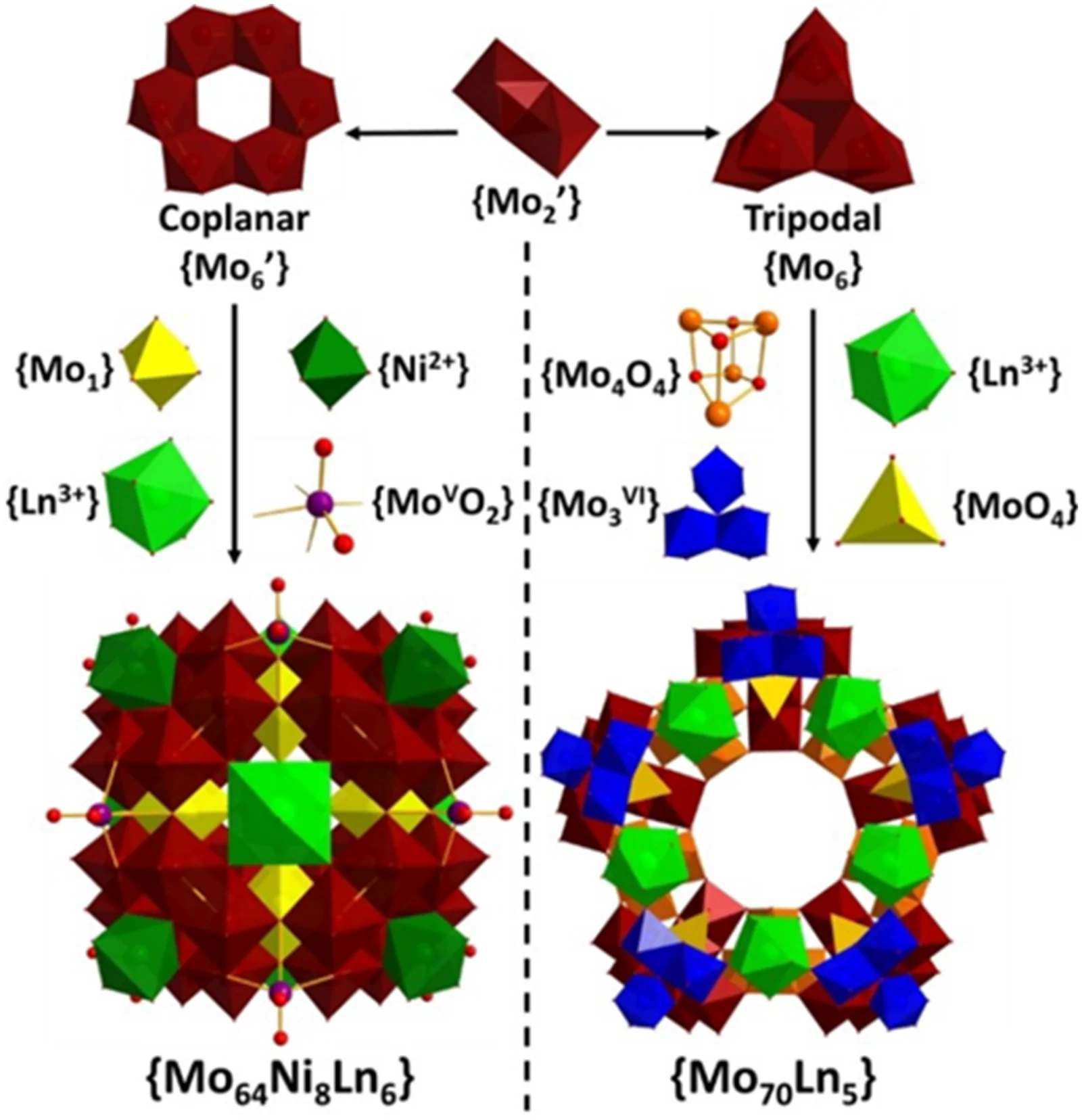
Schematic view of {Mo_64_} and {Mo_70_} formation, with assembly of secondary BB's from an initial pool of {Mo_1_} – yellow, {Mo_2_-er} – maroon, {Ln^3+^} – light green, {Ni^2+^} – dark green and {Mo^V^O_2_} – purple.^39^

Recent work shows that larger Mo red clusters are mostly composed of two well-defined BBs, hexagonal {Mo^V^_6_} and tripodal {Mo^V^_6_} (Fig. 5), each containing three Mo–Mo bonded dimers. Interestingly, hexagonal {Mo^V^_6_} and tripodal {Mo^V^_6_} are exactly two half of the ε-Keggin isomer {Mo^V^_12_},^49^ which is fully reduced and also the smallest Mo red structure.^54^ We recently^39^ conducted exploration into Mo reds and discovered the {Mo_64_} supercube and {Mo_70_} star. The {Mo_64_} supercube represents a hetero-metal POM, containing 64 Mo atoms along with eight Ni and six Ln atoms. Its structure is composed of five distinct geometric layers formed by five different types of BBs. The primary cubic layer comprises of 48 Mo^V^ atoms, distributed across eight coplanar {Mo^V^_6_} BBs, creating a truncated cuboctahedral geometry. Secondly, each {Mo^V^_6_} BB is capped by a {NiO_3_(H_2_O)_3_} moiety, with eight such groups defining the overall molecular cubic geometry. Thirdly, a cuboctahedron layer composed of 12 {Mo^VI^} sites positioned beneath the cubic layer edges, serving as bridges between the coplanar {Mo^V^_6_} units. The fourth octahedral layer contains six {Ln^III^(H_2_O)} centres distributed across central positions of each face of the {Ni_8_} cube. {Ln^III^(H_2_O)} bridges four coplanar {Mo^V^_6_} BB and four {Mo^VI^} units on each cube face. The final geometric layer consists of 4 {Mo^V^O_2_} BBs disordered across 12 positions over the cube edge centres, each possessing an occupancy of 0.33 (Fig. 5).

The {Mo_70_} star is composed of two types of BBs, five {Mo_10_} units and five {Mo_4_} units, linking in an alternating fashion. The {Mo_10_} unit consists of the aforementioned tripodal {Mo^V^_6_} BB with a {Mo^VI^_3_} addenda, templated by a {Mo^VI^O_4_} unit (Fig. 5). The {Mo_4_} = {Mo^V^_2_Mo^VI^_2_} BB containing a Mo–Mo bond has a cubane structure. The {Mo_70_} star shaped cluster base has also been viewed as the main BBs in the construction of the largest Mo red, {Mo_240_} and its analogue structures {Mo_250_} and {Mo_260_}.^35^

The {Mo_240_} dodecahedra is a cage structure of 240 nuclearity, possessing 12 open pentagonal windows. It consists of 20 tripodal {Mo^V^_6_} and 30 {Mo^V^_2_Mo^VI^_2_} units, where these assemble into the same star motif as the {Mo_70_}^39^ cluster, however instead of being stabilised and templated by {MoO_4_}, the tripodal units are templated by SO_3_^2−^ or SO_4_^2−^ anions. When the tripodal BB is templated by SO_3_^2−^ it is found at a chassis position, whilst in the case of SO_4_^2−^ the anion can be found in its centre.

## 3. Electronic structure

### 3.1. Origin of characteristic colours

The family of giant POMos is notable both for their exceptional molecular size and their distinctive colour, which arise from their reduction state as well as the distribution of reducing electrons. As previously discussed, Mo-based POMs possess transferrable BBs that assemble into the larger POM architectures through specific, reproducible joining operations. The resulting BB configurations and their reduction state directly dictates the colour produced by the POM species. The MB wheels exemplify this relationship, where electron delocalisation along the structural backbone motif gives rise to the characteristic blue hue.

**Fig. 6:**
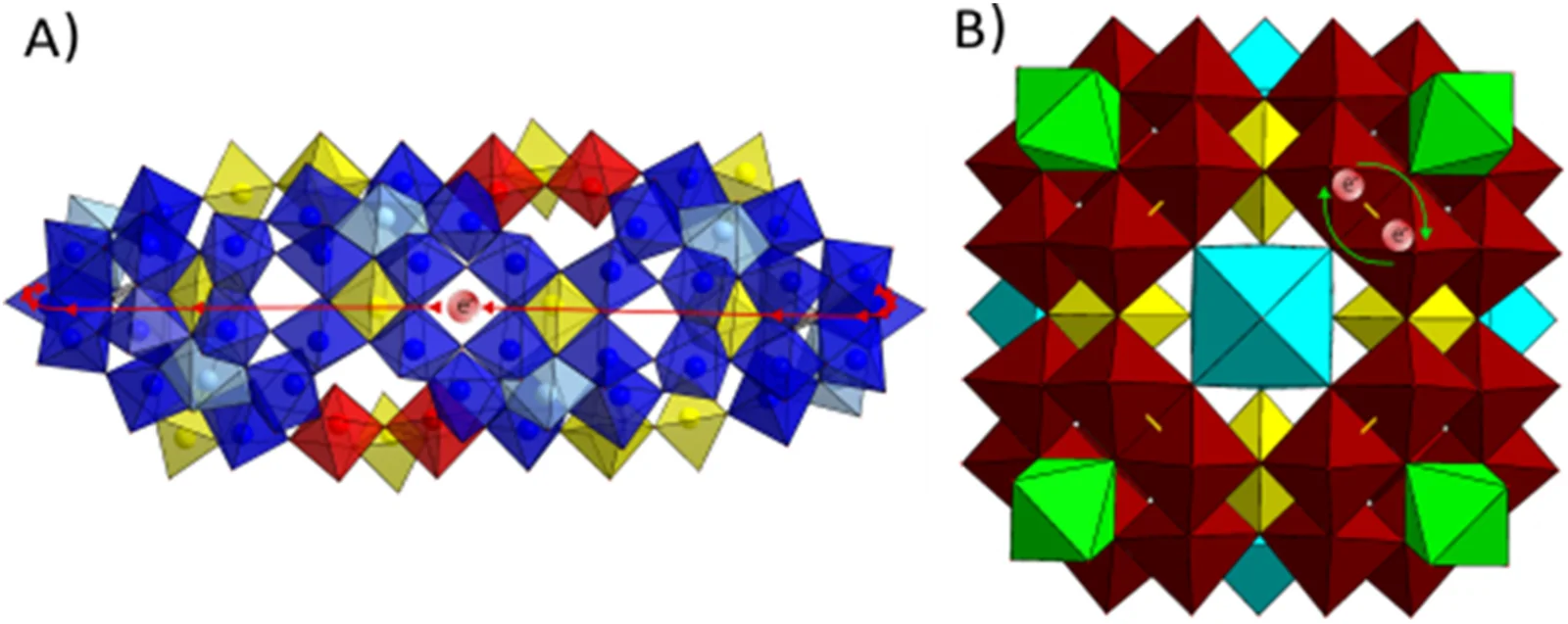
(A) Delocalisation of reducing e-across backbone of MB POMs. e-Found in double-cubane compartments between which electron hopping occurs, producing the characteristic blue. (B) Localisation of reducing electrons across {Mo_2_-er} BB producing characteristic red.

Muller *et al.*^58^ observed that two delocalised 4d electrons are found within the {Mo_5_O_6_} double-cubane compartments of the backbone. It is at these positions that we observe “electron hopping”, from one compartment to another, undergoing intervalence charge transfer (IVCT) of Mo^V^ → Mo^VI^, yielding the deep blue colour. In contrast, the more highly reduced Mo brown Keplerate clusters exhibit localised reducing electrons on {Mo^V^_2_}/{Mo_2_-er} dimers with direct Mo–Mo bonds, resulting in their distinctive brown coloration (Fig. 6).

### 3.2. Charge distribution

The reducing electrons possessed by a given POM can be delocalised, localised, or a mixture for a given cluster (Table 2). However, it can be possible to gain further insight to more probable positions for the delocalised electrons. This has been highlighted in our recent work producing compressed MB rings, where it was observed that specific positions within the MB POM display a greater probability of possessing the reducing electrons.^34^ The distribution of electron density can also be visualised using density functional theory (DFT) or by evaluating using BVS calculations.

The charge distribution of the {Mo_102_} cage explained by Muller *et al.*,^28^ who observed that it is a mixed valence species possessing 36 Mo(4d) electrons. Of these electrons, 30 are localised on the 30 {Mo_1_-b} units, which act as bridges between pentagonal {Mo_6_} BBs. The remaining six electrons are delocalised across the Mo centres of the 12 pentagonal {Mo_6_} BBs. Normally a system having a high Mo^V^ : Mo^VI^ ratio would produce localised {Mo^V^_2_} bonds, and a low ratio would display delocalised reducing electrons. Despite the large number of Mo^V^ centres, which might suggest the formation of Mo^V^–Mo^V^ bond {Mo^V^_2_}, the {Mo_102_} assembles without such bonds. This phenomenon arises from the unique structure of the cluster where no symmetrically comparable Mo–Mo pairs exist.

The distribution of charge was also explained by Bo *et al.*,^59^ where the molecular and electronic structures of two Keplerate's, K-{Mo_132_} and {W_72_Mo_60_}, were investigated using density functional theory (DFT). It was found that the oxo bands were filled while metal bands empty; the HOMO band is composed of Mo^V^ atoms present in the 30 {Mo^V^_2_} units in K-{Mo_132_}. The hetero-metallic species {W_72_Mo_60_} presents a larger HOMO–LUMO gap than K-{Mo_132_}, suggesting it possesses greater stability. Furthermore, charge distribution analysis determined that the central metal atoms of the pentagonal {Mo_6_} BBs are most positively charged, whilst their terminal oxygens are least basic.

Recently, Chen *et al.*^33^ discovered the first Mo green species, a capped wheel C-{Mo_132_}. This species consists of both pentagonal {Mo_6_} BBs and edge-sharing {Mo_2_-er} spacers. It was observed that the reducing electrons are distributed across the double-cubane compartments of the backbone belt, with additional localised electrons within the 10 Mo^V^–Mo^V^ units. DFT studies comparing K-{Mo_132_} and C-{Mo_132_} confirmed the delocalised electron distribution in C-{Mo_132_}, whilst reinforcing the localised nature of reducing electrons in K-{Mo_132_}. Notably, the UV/vis absorption peak of C-{Mo_132_} arising at 750 nm suggests that the number and location of reducing electrons play critically influence on the colour of the products.^33^

In addition to predicting the locations of reducing electrons, it is also possible to estimate the positions of protons within the cluster. These protons are typically bound to either bridging or terminal oxo groups. Terminal oxo species may accommodate up to two protons, which then exist as aqua ligands. Bridging oxo groups generally form hydroxo ligands, though a tertiary oxo group may become protonated if it participates in the formation of a double-cubane compartment. Protons may also share positions with metal or heteroatom in partial occupancy.

## 4. Properties

### 4.1. Self-assembly and auto-catalysis

The formation of MB POMs occurs *via* a complex autocatalytic self-assembly process which has previously been suggested to follow either fixed BB arrangement or internal templating mechanism. To gain deeper insight into the self-assembly processes, Cronin *et al.*^60^ investigated the formation mechanism of a MB wheel under flow conditions, using a system that allowed precise control of key parameters such as pH, molybdate concentration, and reducing agent levels, all critical for successful MB POM formation. By establishing an off-equilibrium system, it was possible to regulate and monitor the degree of POM cluster reduction. Single crystals could form only at an appropriate flow rate: too low and the reduced molybdate concentration was insufficient for crystallisation, whilst too fast, the system would become over reduced. Analysis of the resulting product revealed a lacunary {Mo_154_} structure, specifically a {Mo_150_} wheel, formed with the loss of two {Mo_2_} units.

**Fig. 7:**
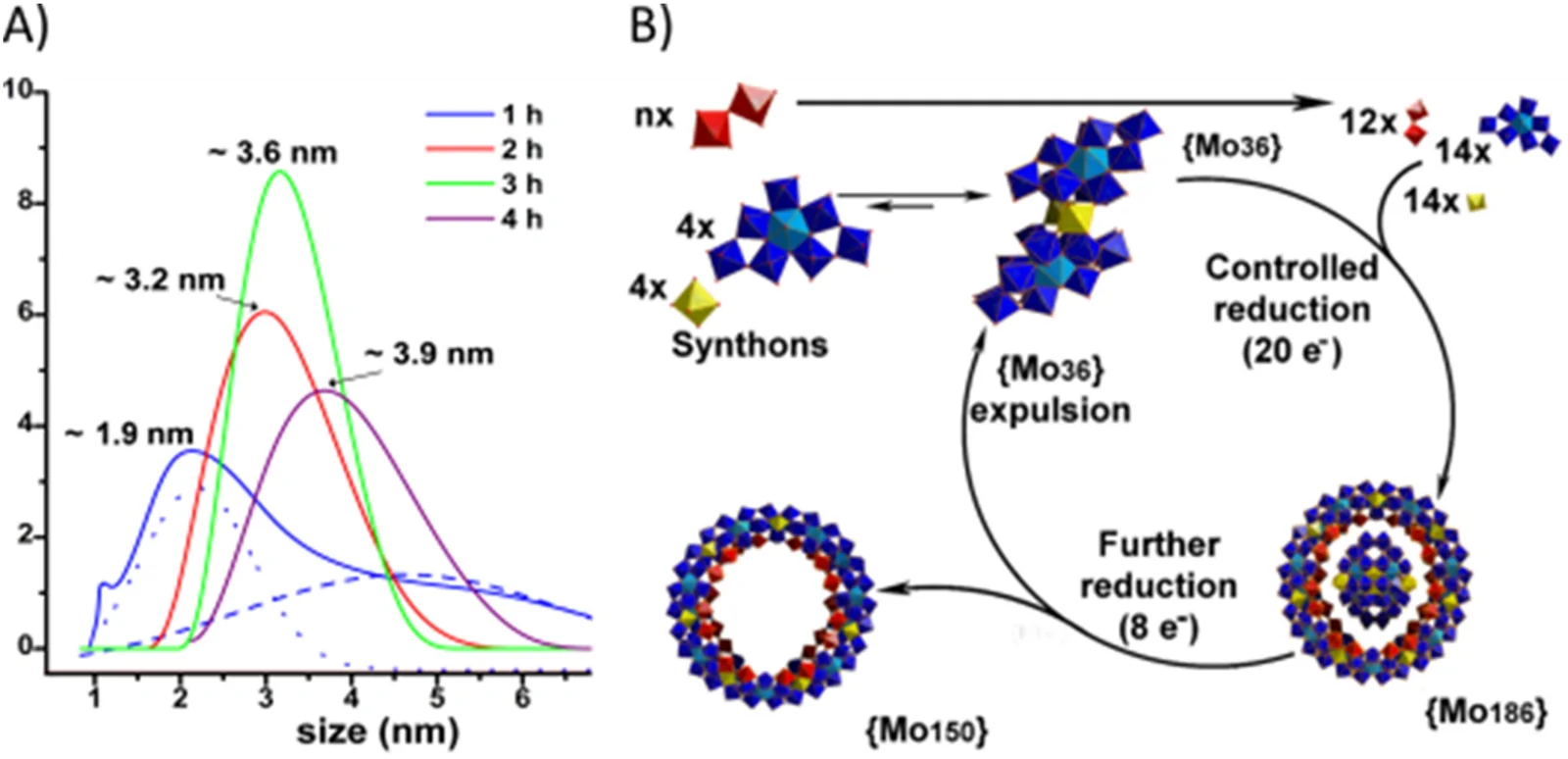
Self-assembly and auto-catalytic formation of giant MB wheels *via* the generation of the {Mo_36_} templated complex. (A) DLS plot displaying evolution of particle size for a molybdate solution over time to elucidate a kinetic and mechanistic model for MB formation. (B) The autocatalytic cycle through which MB wheels form.^60^

The product {Mo_36_@Mo_150_} obtained from the flow system contains an internal {Mo_36_} guest unit, suggesting that the {Mo_36_} unit templates the formation of {Mo_154_} (Fig. 7). Based on these insights into the {Mo_154_} formation mechanism, it can be inferred that, to promote the formation of the giant wheel, the reaction conditions should also facilitate the initial formation of {Mo_36_}. This then templates the assembly of {Mo_154_} and subsequently drives an autocatalytic cycle for further giant wheel production. The autocatalytic nature of {Mo_154_} formation was confirmed using Dynamic Light Scattering (DLS), which provided evidence for the self-assembly process. This technique allowed for identification of the autocatalytic process, where an embedded template proceeds to be transferred from one species to another in response to changes in reduction potential and solution pH. Due to the fast kinetics of molybdate systems, replicating species are capable of emerging and thus POMo clusters.

It has also been observed that the self-assembly process can yield multiple species in solution. Kogerler *et al.*^61^ undertook a spectroscopic investigation into the formation of {Mo_154_}, {Mo_102_} and {Mo_368_}. It was observed that these species possess an ideal degree of reduction and pH to undergo crystallisation but furthermore that the aging of samples allowed for crystallisation of species which co-exist in solution.

With the formation mechanism and common BBs in mind, it is important to then have recognition of the common architectures adopted by MB POMs. These are the aforementioned rings {Mo_154_},^25^ balls {Mo_102_}^28^ and cages {Mo_248_}.^27^ These frameworks can be considered to be hierarchical, where the {Mo_154_} is the archetypal wheel, but the faces of the ring can be closed, giving rise to clusters such as C-{Mo_132_}.^33^ Alternatively, the rings can be compressed, resulting in elongation of the ring along one axis {Mo_108_}.^34^ These clusters all possess common BBs, notably the {Mo_6_} pentagon and the supplementary {Mo_1_-b} BB.

The formation mechanism of POMos has also been further investigated by Chen *et al.*^62^ who studied the electrocatalytic self-assembly routes of MB POMs using an aqueous flow system coupled with *in operando* Raman characterisation. Their experimental set-up initially tracked the transformation of simple molybdate salts, MoO_4_^2−^, into Mo_7_O_24_^6−^, as evidenced by Raman spectral evolution. The initial MoO_4_^2−^ signal at 897 cm^−1^ decreased over time while a new peak emerged at 940 cm^−1^ – corresponding to the Mo_7_O_24_^6−^ formation. As the system was continually acidified, this peak shifted to 948 cm^−1^ due to the protonation of the molybdate species. Further system evolution produced new peaks at 902 cm^−1^ (*ν*_asym_) and 983 cm^−1^ (*ν*_sym_), with the main peak shifting to 954 cm^−1^. These spectral changes indicated {Mo_36_} formation, later confirmed by single crystal analysis. The study was further expanded to incorporate electrochemical control, using both {Mo_36_} and Na_2_MoO_4_ as starting reagents to facilitate {Mo_154_} formation. During electrochemical reduction, the characteristic {Mo_36_} Raman peaks diminished while new vibrations appeared at 218, 320, 485 and 528 cm^−1^, corresponding to Mo^V/VI^ vibration modes in {Mo_154_}. When H_2_SO_4_ was used as the acid, the major product shifted to {Mo_102_}. Raman contour maps revealed a distinct self-assembly pathway, implicating SO_4_^2−^ in forming key intermediary species for {Mo_102_} generation. Complementary ESI-MS studies provided additional mechanistic insights into the self-assembly process. Oxidation of {Mo_36_} solutions yielded [NaH_16_Mo^VI^_5_O_24_]^−^ (*m*/*z* = 902.4), an oxidised pentagon unit, which upon reduction transferred into [H_18_Mo^VI^_4_Mo^V^O_24_]^−^ (*m*/*z* = 881.4). This pentagon unit is an important, transferable BB across MB POMs. Complete consumption of {Mo_36_} during further reduction was confirmed by the disappearance of its ESI-MS signal.

### 4.2. Functionalisation

**Fig. 8:**
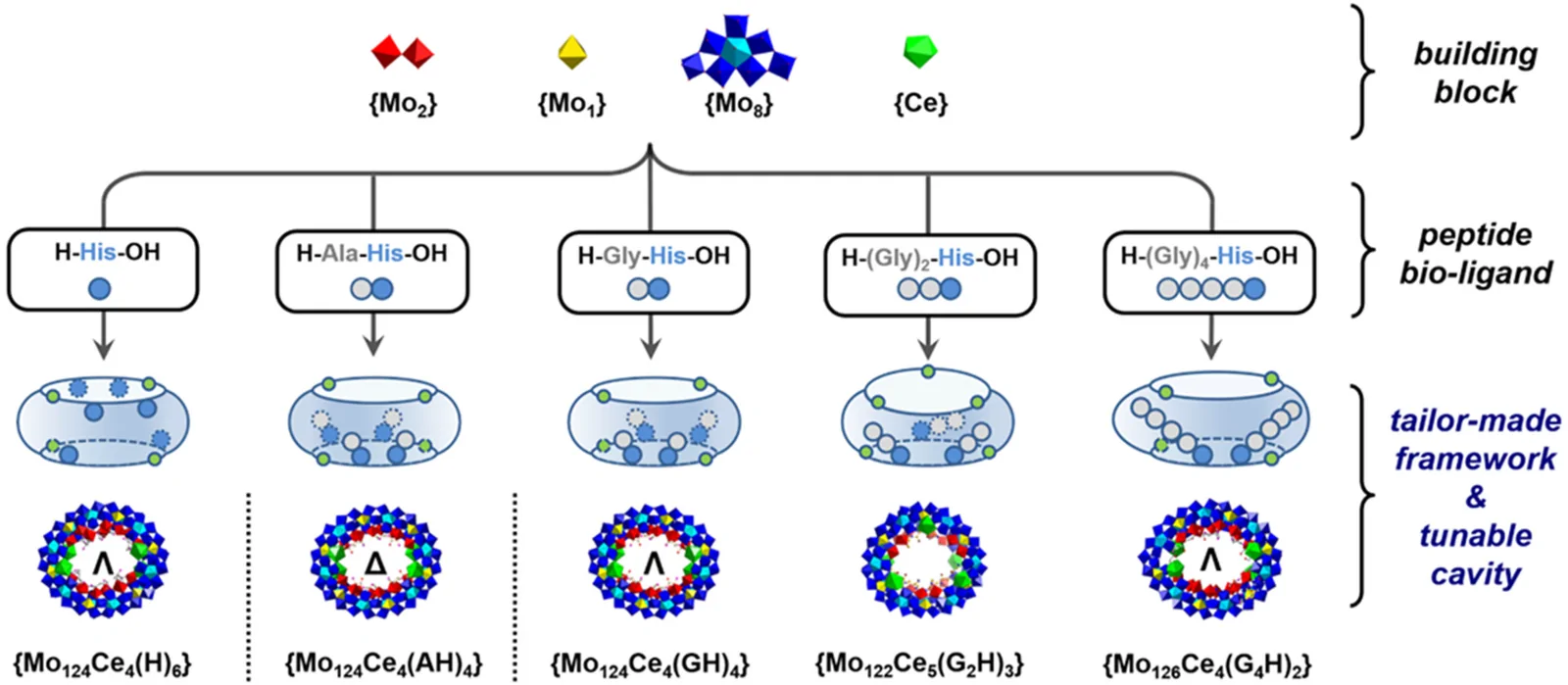
Schematic view of peptide directed self-assembly process of chiral MB wheels. Drums indicate the mounting points of peptides and positions of Ce^3+^ inducing chirality of the cluster.^41^

A series of studies has explored the functionalisation of MB wheels through the incorporation of organic ligands. Initial work demonstrated the successful grafting of amino acids such cysteine and tyrosine onto {Mo_154_} architecture while preserving its wheel-like structure. Subsequent research expanded this approach by modifying a {Mo_150_} wheel.^30^ In this system, several l-ornithine (l-Orn) ligands were successfully grafted onto {Mo_2_-c} BBs, with their tails oriented toward the cluster interior. Further development expanded this strategy by incorporating different amino acids into the cavities of both pristine and Ln-substituted MB clusters, generating a family of organically functionalised MB clusters. As the number of successfully grafted amino acids increased, the scope of organic modifications was broadened to include peptide sequences, demonstrating the versatility of MB clusters as platforms for hybrid inorganic–organic assemblies (Fig. 8).

The peptide sequence serves as multivalent ligands that modulate the internal cavity, arrangement, and symmetry of Ln-substituted MB wheels by directing their self-assembly process. Several oligopeptides were investigated as structure directing ligands and were successfully grafted into the Ln MB wheel cavities to create enzyme-like active sites (Fig. 8). The research demonstrates the capability for organic functionalisation of MB wheels and establishes peptides as versatile, multifunctional ligands in POM chemistry. The binding capacity for both peptide and amino acids depends on the number of Ln substitution sites within the cluster. For instance, the {Mo_124_Ce_4_} wheel can accommodate six amino acids but only three to four peptides, reflecting the larger steric demands of peptide ligands. Notably, the mounting points differ between these ligand types: the amino acids bind to {Mo_2_-c} units on both sides of the cluster, while peptides exclusively attach to {Mo_2_-c} units on one side of the wheel.

Another important aspect to consider during MB synthesis is the generation of chiral POM clusters. The chiral functionalisation of wheel and cage-shaped MB POMs is particularly durable, as these structures can provide confined chiral environment for application in asymmetric catalysis or chiral recognition, and even as artificial protein mimics. However, constructing chiral POM frameworks from assemblies comprising hundreds of atoms presents substantial synthetic difficulties. Cronin *et al.*^43^ demonstrated the stereo-selective synthesis of chiral MB POMs using amino acids with lanthanides acting as symmetry breaking elements. When only lanthanides as symmetry breakers, the synthesis always produces a racemic mixtures of both enantiomers. Pure enantiomers can be selectively produced by introducing amino acids and peptide ligands. Several compounds were synthesised, based on the {Mo_124_Ce_4_} framework, which give rise to an inherently chiral, Δ or Λ, configuration, depending on the configuration of the chiral amino acid or peptide.

### 4.3. Manipulation by hetero-species substitution

Synthetic means towards discovery of new MB POMs can be further expanded through elevated and hydrothermal heating. Traditional MB POM synthesis is typically conducted at room temperature due to the potential of reduced Mo^V^ oxidising, coupled with the perception that MB POM species would be unstable and prone to degradation at elevated temperatures. Recent advances in MB POM synthesis have shown that the use of elevated temperatures are required towards discovery of new MB species,^32,34,48^ a method previously associated primarily with the formation of Mo reds, the most reduced family of giant POMo's.

**Fig. 9:**
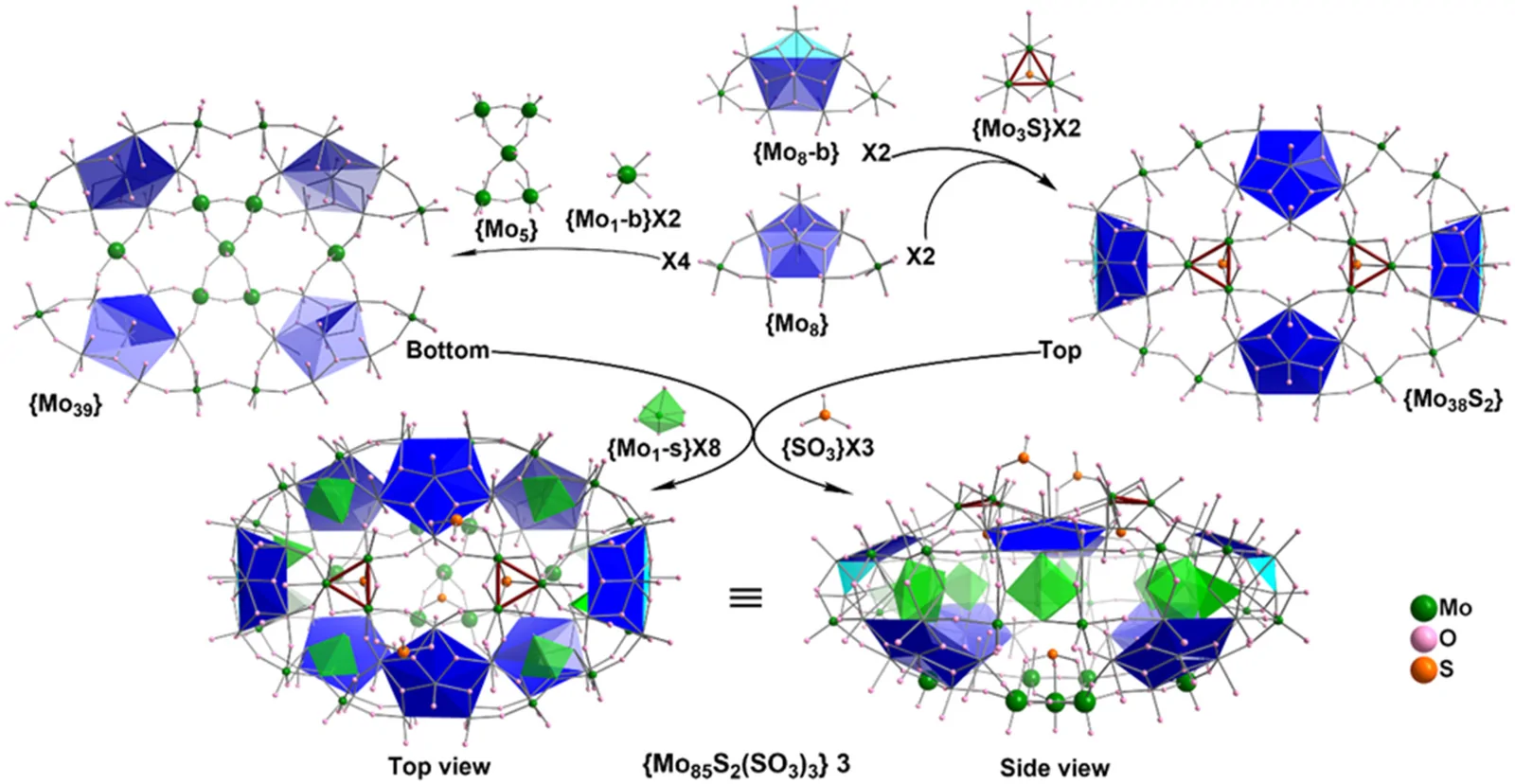
Schematic view of assembly of compressed ring {Mo_85_}. Construction dependent on the formation and assembly of both regular {Mo_8_} and distorted pentagon {Mo_8_-b}, alongside bridging units {Mo_1_-b}, {Mo_5_}, and {Mo_3_S}.^34^

By using hydrothermal conditions, alongside sulphite or sulphate templates, highly compressed MB POM rings can be produced. These rings display compression from 154 to 54 Mo atoms. The ring compression gave rise to new, and rarely observed, BB: a reduced {Mo^IV^_3_} trimer capped with a sulphide group, a distorted {Mo_6_} pentagonal unit that directly participates in backbone formation, and a {Mo_5_} bridging unit that aids in ring capping. The construction of these compressed clusters was significantly facilitated by the *in situ* formation of sulphur-based templates. For instance, the {Mo_54_} ring utilises a SO_4_^2−^ template while the {Mo_58_} ring uses a mixture of SO_3_^2−^ and SO_4_^2−^ templates. The {Mo_85_} ring in contrast employs both SO_3_^2−^ and S^2−^ for its assembly (Fig. 9). Hydrothermal heating has proven instrumental in forming new architectures, enabling not only ring compression but also the emergence of closed shell structures. Notably, a geometric isomer of the classical K-{Mo_132_} Keplerate, the lantern L-{Mo_132_}, was synthesised.

The L-{Mo_132_}^32^ possesses an architecture, resembling a traditional Chinese lantern, features an exterior decorated with {Mo^IV^_3_S} trimers. A further capped wheel C-{Mo_132_} was also produced under similar hydrothermal conditions. The synthesis followed a simple one pot process, resulting in quasi-isomerism of K-{Mo_132_}. This discovery highlights the potential for isomerism in giant POMos, as exemplified by L-{Mo_132_} and C-{Mo_132_}.

The structural diversity of MB POMs arises from the transferrable [(Mo)Mo_5_]/{Mo_6_} BB, serving as a common motif. In classical wheel type architectures, the wheel is generated *via* the assembly of {Mo_6_}, {Mo_2_-c} and {Mo_1_-b} units, however greater diversity in wheel architectures can be achieved *via* substitution of small BB. It is possible to resize wheels *via* Ln substitution of corner sharing {Mo_2_-c} units. Muller *et al.*^37^ demonstrated this by substituting {Mo_2_-c} units with {Pr(H_2_O)_5_} which not only reduces the ring's negative charge, but also alters its curvature due to the smaller size of the Ln ion.

Furthermore, the Ln ions are capable of acting as symmetry breakers,^40^ adjusting the ring curvature and functionality of the internal ring. Ln ions act as symmetry breakers as a consequence of the reduction in oxygen-to-oxygen (O-to-O) distance of adjacent {Mo_6_} units, where traditional {Mo_2_-c} units have a typical inner O-to-O distance of 4.2 A whilst Ln bridged distance is reduced to 2.8 A, compressing the outer O-to-O distance from approximately 7.3 A to 4.2 A – causing the shift in curvature due to reducing O-to-O distances by almost half. A subsequent consequence of the BB replacement is that some of the pentagonal {Mo_6_} units are not used during the self-assembly process, and results in the formation of smaller wheels, such {Mo_128_Ce_4_},^40^ {Mo_120_Ce_6_},^38^ {Mo_100_Ce_6_}^45^ and {Mo_90_Ce_10_}.^31^ As seen by these examples, increasing the Ln content further reduces wheel size. Symmetry changes are evident when comparing {Mo_154_} (*D*_7d_ centrosymmetric) with {Mo_150_La_2_}^63^ (*C*_2h_ centrosymmetric), where two La^3+^ ions are centro-symmetrically related. However, not all partially substituted rings retain centro-symmetry, for example {Mo_128_Ce_4_} and {Mo_120_Ce_6_} are non-centrosymmetric. When the complete substitution, as in {Mo_90_Ce_10_}, restores *D*_5d_ centro-symmetry.

Notably, the fully substituted {Mo_90_Ln_10_}^31^ cluster (where Ln = La, Ce and Pr) represent a unique family of neutral POMs, due to their net ring charge being zero. This distinguishes them from all other giant POMos, which are inherently anionic. Interestingly, when Nd and Sm was used as the lanthanide during synthesis, a second species {Mo_92_Ln_9_} formed. Unlike the fully substituted {Mo_90_Ln_10_}, this cluster possesses a {Mo_2_-er} linker (instead of the original {Mo^VI^_2_} unit), resulting in an incompletely lanthanide-substituted structure.

### 4.4. Supramolecular chemistry

The family of giant POMo's has demonstrated capabilities in forming supramolecular assemblies. A notable example was reported by Cadot *et al.*^64^ who investigated the supramolecular assembly of the giant wheel {Mo_154_} in *n*-octyl-β-glucoside (C8G1). Their study found that {Mo_154_} exhibits a high affinity for C8G1 and undergoes a stepwise assembly process, transitioning from discrete species to vesicles, lamellar aggregates and ultimately nanosheets. This structural evolution was driven by accurately controlling the C8G1 concentration, where increasing the concentration altered the final assembly.

**Fig. 10:**
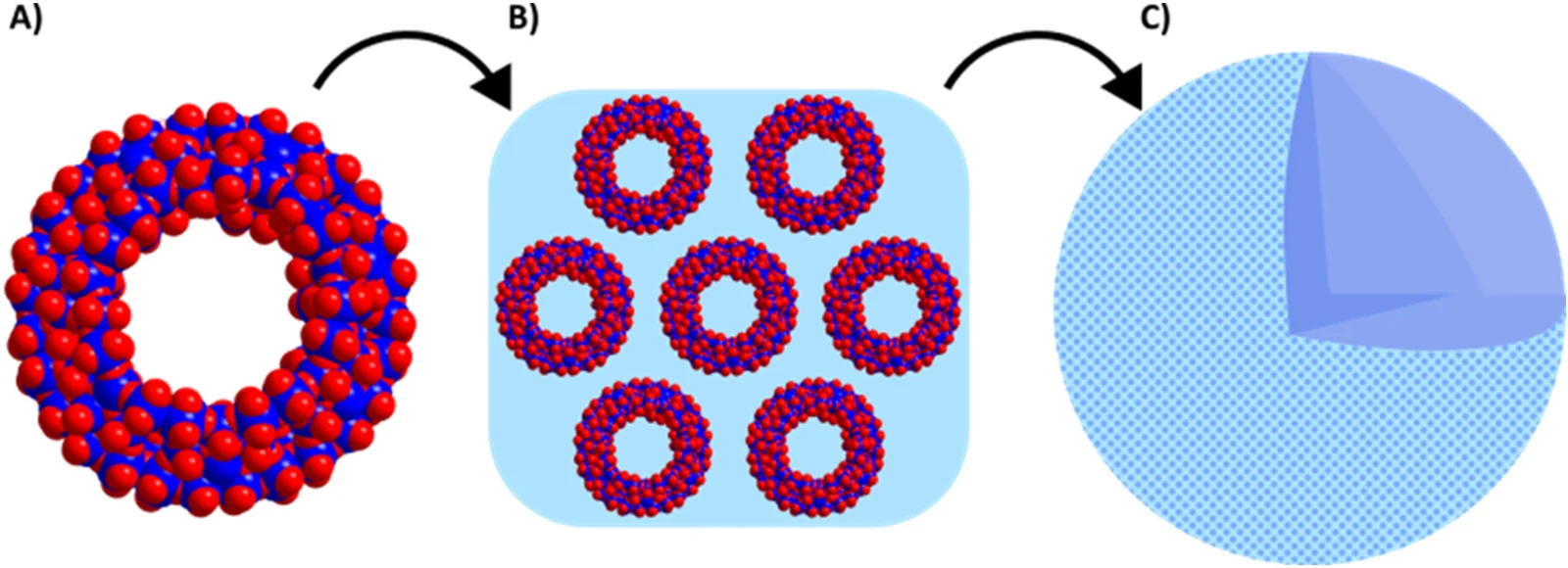
(A) Space fill model of {Mo154} wheel. (B) Ordered assembly of {Mo154} wheels (C) organisation of discrete {Mo154} wheels into a supramolecular vesicle/blackberry assembly.

Muller *et al.*^17^ identified that discrete {Mo_154_} giant wheel molecules can undergo self-assembly to form well-defined, spherical, metal–oxide aggregates. These supramolecular aggregates, termed blackberries, were characterised using dynamic light scattering (DLS) and transmission electron microscopy (TEM), identifying aggregates composed of 1 165 {Mo_154_} clusters. The clusters align in a flat manner, and are uniformly distributed across the vesicle surface, driven by short-range van der Waals attractions and longer ranged electrostatic repulsions. Unlike conventional vesicles, these POM-based assemblies are not stabilised by hydrophobic interaction but instead stabilised due to hydrogen bonding between water molecules encapsulated between the vesicle's interior and the discrete clusters. The vesicle size can be finely tuned by adjusting the solution pH, with lower pH values leading to larger vesicles. Furthermore, addition of electrolytes to the solution can also affect vesicle sizes; for instance, addition of NaCl produce larger assemblies (Fig. 10).

The blackberry assembly is not limited to the {Mo_154_} ring but can also be achieved by MB ball type clusters.^19^ For instance, the substituted MB ball, {M_30_Mo_72_} (M = Fe^III^ or Cr^III^) undergoes a self-recognition-driven assembly process in mixed aqueous solutions containing both species. A slow dimer-oligomerisation process occurs through deprotonation of the neutral species, resulting in the formation of two distinct, homogeneous phases of blackberry structures. Notably, the substituted ball structures do not form mixed species. This selective assembly stems from differences in macroionic charge densities, which direct the gradual formation of dimers and oligomers.

The Keplerate type Mo brown clusters exhibit notable host–guest chemistry properties. Specifically, the K-{Mo_132_} cluster functions as an effective inorganic host due to its tuneable, well-defined structure and internal cavity.^58,59^ The chemical nature of the cavity can be modified through the choice of internal ligands, enabling adjustment between hydrophobic and hydrophilic nature. Common ligand options include acetate, propionate, sulphate, or phosphate groups, which determine the cavity's molecular recognition properties.

## 5. Synthesis

### 5.1. Synthesis of molybdenum blues

MB POMs are synthesised using one pot protocol requiring precise control of reduction degree and pH to selectively produce the desired cluster. The archetypal {Mo_154_} wheel was initially synthesised under various conditions by preparing reduced, acidified molybdate solutions. In the original method, hydrazinium sulphate (0.16 g, 1.2 mmol) was added to a solution of ammonium molybdate (1.12 g, 0.9 mmol) and 50% acetic acid (0.24 mL) under an inert atmosphere. This yielded a blue solution which was then left for crystallisation, requiring two months to yield crystalline solids.^25^ This procedure has since been refined with time, where sodium molybdate (3.04 g, 12.6 mmol) is dissolved in 20 mL water followed by the addition of 2.2 ml of 37% HCl. The pH should be adjusted to 0.7–1.0 and sodium hydrosulphite is added (0.15 g, 0.86 mmol) (Table 3). The solution is degassed with nitrogen for five minutes before being left to crystallise for 24 hours, a significant improvement over the original two-month period.^65^ By then adapting these synthetic conditions, the nuclearity of the giant wheels produced can be controlled – where incremental decreases in pH, towards pH 1.0, allows for the growth of larger giant MB towards a cap of 154 membered rings.

**Table 3:** Synthetic parameters of POMos, covering archetypal, and recent advances, of molybdenum blue, brown and red structures

Notation	Reduction family	pH^a^	Acid used	Reducing agent	Templates/additives	Heating conditions^b^	Crystallisation time	Ref.
{Mo_154_}	Blue	0.7–1.0	37% HCl	Na_2_S_2_O_4_	N/A	RT	24 Hours	65
{Mo_368_}	Blue	NR	0.5M H_2_SO_4_	Na_2_S_2_O_4_	N/A	RT	2 Weeks	24
{Mo_108_}	Blue	1.05	6M HCl	N_2_H_4_·2HCl	Li_2_B_4_O_7_	72 Hours @ 120 °C	72 Hours	34
K-{Mo_132_}	Brown	NR	50% HOAc	N_2_H_6_SO_4_	NH_4_OAc	RT	96 Hours	29
L-{Mo_132_}	Blue	NR	3M H_2_SO_4_	Na_2_S_2_O_4_, N_2_H_4_·2HCl	N/A	72 Hours @ 120 °C	12 hours	32
C-{Mo_132_}	Blue	2.20	1M H_2_SO_4_	Na_2_S_2_O_4_	NaOAc·3H_2_O	48 Hours @ 150 °C	1 Month	33
{Mo_240_}	Red	NR	3M H_2_SO_4_	N_2_H_4_·2HCl	CoSO_4_·7H_2_O	96 Hours @ 120 °C	48 Hours	35
{Mo_64_}	Red	2.80	HClO_4_	N_2_H_4_·2HCl	NiCl_2_·6H_2_O, LaCl_3_·7H_2_O	96 Hours @ 100 °C	96 Hours	39

a pH reported as provided in literature. NR = not reported.

b Crystallisation starts during the heating periods of {Mo_108_} and {Mo_64_} hence heating and crystallisation times match.

The general procedure developed for {Mo_154_} was subsequently extended to other MB wheel structures. The {Mo_176_} cluster was produced by introducing molybdenum chloride in ethanol to a solution of sodium molybdate, followed by acidification to pH 1.4. After four days of crystallisation, this procedure yielded blue tetragonal prismatic crystals.^26^ Further expansion of Mo blue synthetic approaches alternation of reducing agents can promote molecular growth. For instance, acidifying a solution of sodium molybdate with hydrochloric acid, followed by the introduction of ascorbic acid, promotes cluster growth from {Mo_176_} to {Mo_248_}.^27^

As previously mentioned, the MB POMs can undergo structural compression. The transformation from {Mo_154_} to {Mo_108_} is achieved by the introduction of hydrazine dihydrochloride, followed by the addition of lithium tetraborate to a solution of ammonium molybdate. The solution was subjected to prolonged heating at 120 °C for 72 hours. Further compression to 85 membered rings involves acidifying an ammonium molybdate solution with sulphuric acid, reducing it with hydrazine dihydrochloride and sodium hydrosulphite, and finally adjusting pH, a critical step for controlling the cluster formation after heating (140 °C for three days). The system can be compressed further, down to 54 membered clusters through fine-tuning synthetic conditions similar to those used for {Mo_85_} but without heating.^34^

Beyond classical wheel-shaped MB clusters, more complex architectures can be synthesised. The {Mo_102_} ball is prepared by acidifying an aqueous solution of {Mo_132_} with hydrochloric acid, followed by addition of sodium chloride. After stirring, the solution crystallises for three days.^28^ Similarly, the lemon-shaped {Mo_368_} cluster can be synthesised *via* the addition of sodium hydrosulphite to a sodium molybdate solution, which is then acidified with sulphuric acid and left to crystallise for two weeks.^24^

MB architectures are capable of undergoing metal substitution, where Mo atoms can exchange with other transition metals. For example, the {Mo_72_Fe_30_} cluster forms when FeCl_3_ and ammonium acetate are introduced to a {Mo_132_} solution, followed by acidification, sodium chloride addition and heating. This substitution proceeds *via* replacement of {Mo^V^_2_} dimers by octahedrally coordinated Fe^III^ aqua ligands, eventually yielding yellow {Mo_72_Fe_30_} crystals.^66^ These clusters can also be functionalised with hetero-species to produce new modes of bonding and substitution of BBs within the wheel. This is exemplified by the {Mo_90_Ln_10_} series, where corner sharing {Mo_2_-c} BBs are replaced by lanthanides. This can be achieved by utilising high temperature reduction of a mixture containing LnCl_3_·nH_2_O and hydrazine dihydrochloride solutions, which is acidified before sodium molybdate addition. Heating for three – four days yields blue rhombic crystals of Ln substituted wheels.^31^

MB clusters can be controlled through the directed self-assembly utilising anionic templates. A notable example uses l-ornithine as a template, where its electrostatic interactions and hydrogen bonding with MB wheel BBs guide the assembly of wheel-shaped clusters ranging from 124 to 154 Mo atoms. In this approach, l-ornithine is introduced to systems undergoing synthesis like Ln-substituted systems (see above). The process involves Ce^3+^ substituting some corner-sharing {Mo_2_-c} dimers, whilst the l-ornithine binds to remaining dimers, with its side chain buried within the wheel structure, producing a l-ornithine functionalised {Mo_124_Ce_4_} cluster. By maintaining the reaction conditions but decreasing the Ce : Mo ratio, a larger wheel {Mo_150_Ce_2_} forms encapsulating a {Mo_17_} moiety. This methodology was expanded to investigate dual templating using both l-ornithine and {PMo_12_}. Adding phosphomolybdic acid to the standard synthesis procedure as above results in the formation of a {Mo_150_Ce_2_} wheel encapsulating a {PMo_12_} moiety. Control experiments conducted without phosphomolybdic acid and CeCl_3_·7H_2_O yielded two crystallographically independent {Mo_154_} wheels, both featuring l-ornithine functionalisation but with two defect sites (missing two corner sharing {Mo_2_-c} units). Notably, only one of the two wheels encapsulates a {Mo_36_} cluster, while the other remains empty.^44^

### 5.2. Synthesis of further reduced polyoxomolybdates

The synthesis of Mo brown and red clusters follows a similar one-pot approach to MB clusters, requiring precise control of pH and reduction degree. However, Mo red clusters typically require elevated temperatures for successful synthesis. This thermal activation is thought to overcome kinetic barriers, as these species appear to be thermodynamically favoured but kinetically inert.

The classical Mo brown, K-{Mo_132_}, is produced *via* a straightforward procedure involving the addition of hydrazinium sulphate (0.8 g, 6.1 mmol) to a solution of ammonium molybdate (5.6 g, 4.5 mmol) and ammonium acetate (12.5 g, 162.2 mmol) in 250 mL of water. After stirring and acidification with 50% acetic acid (83 mL), the solution is left to crystallise for four days in an open flask. The {Mo_132_} Keplerate structure forms through stabilisation of {Mo_2_-er} dimers by bidentate acetate ligands, generating {Mo^V^_2_O_4_(OOCCH_3_)^+^} BBs. This assembly additionally requires pentagonal {MoO_7_} units, whose formation is controlled by the Mo^V^ : Mo^VI^ ratio. Notably, insufficient reduction (low Mo^V^ : Mo^VI^ ratio) favours the formation of MB clusters instead.^29^

Higher Mo^V^ : Mo^VI^ ratios promote the formation of more reduced species, particularly Mo red clusters. The ε-Keggin represents the smallest member of the Mo red family, produced by adding ammonium molybdate tetrahydrate (3.36 g, 2.72 mmol) to 250 mL water. Nickel acetate (11.25 g, 45.2 mmol) is also added to the molybdate solution and then 40 mL of 50% acetic acid was added. Hydrazinium sulphate (620 mg, 4.76 mmol) was then added with stirring before the solution is stirred for 10 minutes and then heated for three days at 65 °C, yielding thin, brown plates. The inherent instability of naked ε-Keggin structure is mitigated by *in situ* binding of Ni^II^(H_2_O)_3_. This stabilisation occurs through complementary electronic interactions: the nucleophilic ε-Keggin, couples with electrophilic Ni^II^(H_2_O)_3_ species, effectively capping window of the coplanar {Mo^V^_6_} unit.^54^

The {Mo_37_} cluster, formerly classified as a Mo brown but more correctly identified as a Mo red, is produced by combining ammonium molybdate, ammonium chloride and hydrazinium sulphate in water, followed by acidification with acetic acid. The solution is then heated at 100 °C for two hours, yielding brown crystals after a two-week crystallisation period. The assembly mechanism is thought to proceed *via* initial formation of the α-Keggin anion, which undergoes reduction-induced isomerisation to form the ε-Keggin core. This highly nucleophilic ε-Keggin core is subsequently stabilised by binding with {Mo^VI^_4_O_3_} groups. Further reduction enhances the nucleophilic character of these [Mo^VI^_4_O_3_] groups, enabling their interaction with and incorporation of the outer {Mo_10_} and {Mo_11_} structural fragments.^52^

The ε-Keggin and its two fragments coplanar and tripodal {Mo^V^_6_} appear to be critical in generating larger Mo red clusters. The star-shaped {Mo_70_} cluster is produced by dissolving sodium molybdate and nickel chloride in water, followed by acidification with perchloric acid. Subsequently, solutions of LnCl_3_·*n*H_2_O and hydrazine dihydrochloride are introduced to the acidified molybdate solution before pH adjustment. The solution is then heated for four days at 100 °C. During this process, {Mo^V^_2_} assembles into tripodal {Mo^V^_6_} arrangements stabilised by {Mo^VI^O_4_} templates, ultimately forming the {Mo_70_} star structure.^50^ Notably this star motif resembles the pentagonal window openings found in the {Mo_240_} dodecahedral cluster. The {Mo_240_} cluster requires a distinct synthetic approach: solutions of ammonium molybdate and cobalt sulphate are combined with hydrazine dihydrochloride. Upon developing a blue colouration, the solution is acidified with sulphuric acid and heated at 120 °C for four days, yielding dark red polyhedral crystals. This {Mo_240_} cluster demonstrates good stability, evidenced by its ability to form stable tetrabutylammonium (TBA) salt. The TBA-{Mo_240_} derivative is prepared by mixing dilute aqueous {Mo_240_} with TBA solution, followed by centrifugation and washing to yield a brown precipitate.^35^

The {Mo_64_} supercube represents a particularly unusual Mo red structure composed of coplanar {Mo^V^_6_} BBs, we previously discovered. This cluster is synthesised by acidifying a solution of sodium molybdate and nickel chloride with perchloric acid, followed by addition of LaCl_3_·7H_2_O. Subsequent reduction with hydrazine dihydrochloride precedes heating at 90 °C for two hours, after which the pH is carefully adjusted to 2.8 before final heating at 100 °C for four days. The assembly process initiates with ε-Keggin formation, stabilised by Ni^2+^ ions, with the presence of Ln^3+^ (Ln = La, Ce, Pr) playing a critical role in directing the molecular growth pathway. In the absence of Ln^3+^, the system preferentially forms nickel-stabilised ε-Keggin cluster [{Ni(H_2_O)_3_}_4_Mo^V^_12_O_40_H_12_], demonstrating how Ln^3+^ specifically templates the supercube architecture. Careful control of La^3+^ concentration is essential, as excess amounts promote formation of {La_3_[LaMo_12_]} and La_2_O_3_·7MoO_3_ side products, highlighting the delicate balance required in this synthetic system. This transformation from ε-Keggin to supercube structure exemplifies the precise control achievable in directing POM self-assembly through careful manipulation of metal ion additives.^39^

## 6. Characterisation

With a range of synthetic methods towards producing MB POMs established, the next critical step is their efficient separation and characterisation. Common separation techniques include filtration or mechanical separation of crystals from the mother liquor, followed by rinsing with ice cold water. For comprehensive characterisation, single crystal X-ray diffraction (SCXRD) should be performed first, followed by complementary techniques such as bond valence sum (BVS) calculations, thermogravimetric analysis (TGA), elemental analysis and UV-Vis-NIR spectroscopy.

In addition to conventional filtration and mechanical separation, gel electrophoresis has also shown the capability to separate POMo species in solution. This method is particularly useful due to the varying mass, size, and charge of POMo species present in solution. For instance, we have tested the use of gel electrophoresis on the separation of POMo species {Mo_154_} and Keplerate K-{Mo_132_}.^67^ It was observed that the spherical K-{Mo_132_} cluster exhibit greater mobility than the wheel shaped {Mo_154_}, likely due to its higher charge. Furthermore, gel electrophoresis effectively isolated the {Mo_154_} giant wheel from mixtures containing other smaller POMs, demonstrating its utility in POM purification.

Following crystal separation/isolation and scrutiny under microscope, SCXRD can be carried out. It is important that a crystal of appropriate size is chosen – no bigger than the diameter of the X-ray beam used because of heavy absorption issue of POMos. Powder XRD can supplement SCXRD for phase purity assessment, though its broad peaks often complicate data interpretation. After obtaining crystallographic data, refinement using software SHELX on platforms such as Olex2 or WingX, coupled with BVS calculations, is essential. BVS involves analysing bond lengths to estimate the oxidation state of the Mo centre by summing the bond valences of its Mo–O interactions. By repeating this process for every Mo atom in the cluster, the number of reduced metal centres can be determined. This process can also be applied to O atoms to approximate proton distribution through the cluster. The BVS results can be experimentally verified by performing redox/cerimetric titrations. By titrating the POMo solution with cerium(iv) sulphate, the reduction potential of the solution can be monitored, providing the number of reducing electrons and thus the number of reduced Mo centres in the cluster. Additionally, CHN elemental analysis quantifies H content, confirming protonation sites. To determine the number of crystalline water molecules present, TGA is typically conducted from 0 to 200 °C, where the first weight loss event corresponds to the crystalline solvent evaporation, followed by ligand water molecule loss.

Last but not least, inductively coupled plasma optical emission spectroscopy (ICP-OES) or scanning electron microscopy coupled with energy dispersive spectroscopy (SEM-EDS) should be used to determine the elemental composition, most importantly for counter cations. By using these techniques, the composition of the structure will be determined, aiding the structure refinement accuracy. If heteroatoms are present, their oxidation states can be experimentally derived using techniques such as X-ray photoelectron spectroscopy (XPS), further validating the refined structure.

## 7. Outlook

The designed synthesis of giant molybdenum polyoxometalates is a complex process requiring acute understanding of the directed self-assembly process. These processes are directed by the pH, degree of reduction, temperature of reaction and crystallisation times whilst further functionalisation by introduction of hetero species allows for the emergence of related architectures for a given POM framework. By exploiting the redox properties of the POMs, these species display a range of applications across biosensing, catalysis and drug delivery where further exploration and fine-tuning of the species will allow for greater efficiency a wider breadth of applications for giant polyoxomolybdates. To engineer new giant polyoxomolybdates, synthetic methods should continue to be innovated on by methods such as introducing organic ligand moieties, utilisation of anionic templates, and also the use of a heating step to drive the generation of rarely utilised building block such as {Mo^V^_2_-er} and distorted {Mo_8_-b}.

Coupled with new synthetic methods, a digital chemistry approach can aid chemists in exploring the vast chemical space occupied by giant POMos. By using an automated synthesis platform, the way tedious one factor at a time experimental approaches are conducted can be revolutionised using modern statistical methods, and these can be coupled with the possibility of also screening a hardware constrained array of reagents discovering new reactivity. Coupling the automated approach with the introduction of a machine learning algorithm allows for further improvements in the efficiency of exploration of the chemical space, where human biases are eliminated allowing for unconventional syntheses to be conducted, allowing for potential discovery of new POMo assemblies. Here the use of digital control, including precise control of redox, pH, concentration, and other ligands whilst integrating real time sensor data such as spectroscopic inputs and the observation of crystallisation is set to revolutionise the control of POM-assembly and understanding of mechanism.

## Author contributions

The manuscript was written through contributions of all authors. All authors have given approval to the final version of the manuscript.

## Conflicts of interest

There are no conflicts to declare.

## Data Availability

No primary research results, software or code have been included, and no new data were generated or analysed as part of this review.
